# Developing and evaluating communication strategies to support informed decisions and practice based on evidence (DECIDE): protocol and preliminary results

**DOI:** 10.1186/1748-5908-8-6

**Published:** 2013-01-09

**Authors:** Shaun Treweek, Andrew D Oxman, Philip Alderson, Patrick M Bossuyt, Linn Brandt, Jan Brożek, Marina Davoli, Signe Flottorp, Robin Harbour, Suzanne Hill, Alessandro Liberati, Helena Liira, Holger J Schünemann, Sarah Rosenbaum, Judith Thornton, Per Olav Vandvik, Pablo Alonso-Coello

**Affiliations:** 1 Population Health Sciences, University of Dundee, Kirsty Semple Way, Dundee, DD2 4BF, UK; 2 Norwegian Knowledge Centre for the Health Services, Postboks 7004 St Olavs plass, Oslo, 0130, Norway; 3 National Institute for Health and Clinical Excellence, Level 1A, City Tower, Piccadilly Plaza, Manchester, M1, 4BT, UK; 4 Academic Medical Centre of the University of Amsterdam, Meibergdreef 91105 AZ, Amsterdam, The Netherlands; 5 McMaster University Health Sciences Centre, 1280 Main Street West, Hamilton, ON, L8S 4K1, Canada; 6 Department of Epidemiology, Lazio Regional Health Service, Italian Cochrane Network, Via di Santa Costanza, 53, Rome, 00198, Italy; 7 Scottish Intercollegiate Guidelines Network Delta House, 50 West Nile Street, Glasgow, G1 2NP, UK; 8 Australian National University, Canberra, Australia; 9 The Italian Cochrane Centre, 14/D, Milan, Italy; 10 The Finnish Medical Society Duodecim, PO Box 713, Kalevankatu 11 AFIN-00101, Helsinki, Finland; 11 University Medical Centre, Hugstetter Strasse 55, Freiburg, 79106, Germany; 12 Iberoamerican Cochrane Center, Biomedical Research Institute Sant Pau (IIB-Sant Pau), Sant Antoni María Claret 171, Barcelona, 08041, Spain

**Keywords:** Guidelines, Recommendations, Communication, Presentation formats

## Abstract

**Background:**

Healthcare decision makers face challenges when using guidelines, including understanding the quality of the evidence or the values and preferences upon which recommendations are made, which are often not clear.

**Methods:**

GRADE is a systematic approach towards assessing the quality of evidence and the strength of recommendations in healthcare. GRADE also gives advice on how to go from evidence to decisions. It has been developed to address the weaknesses of other grading systems and is now widely used internationally. The Developing and Evaluating Communication Strategies to Support Informed Decisions and Practice Based on Evidence (DECIDE) consortium (http://www.decide-collaboration.eu/), which includes members of the GRADE Working Group and other partners, will explore methods to ensure effective communication of evidence-based recommendations targeted at key stakeholders: healthcare professionals, policymakers, and managers, as well as patients and the general public. Surveys and interviews with guideline producers and other stakeholders will explore how presentation of the evidence could be improved to better meet their information needs. We will collect further stakeholder input from advisory groups, via consultations and user testing; this will be done across a wide range of healthcare systems in Europe, North America, and other countries. Targeted communication strategies will be developed, evaluated in randomized trials, refined, and assessed during the development of real guidelines.

**Discussion:**

Results of the DECIDE project will improve the communication of evidence-based healthcare recommendations. Building on the work of the GRADE Working Group, DECIDE will develop and evaluate methods that address communication needs of guideline users. The project will produce strategies for communicating recommendations that have been rigorously evaluated in diverse settings, and it will support the transfer of research into practice in healthcare systems globally.

## Background

Health professionals, patients, policymakers, and the public aspire to making healthcare decisions on the basis of the best available research evidence [1-6]. However, experience shows that this frequently is not achieved [7-10]. Reasons for this deficiency include the overwhelming amount of research literature, the sometimes contradictory nature of this literature, and presentations that are difficult for non-researchers to understand [11,12]. The complexity of the problems encountered both in patient encounters (*e.g.*, multimorbidy) and health policy decision-making mean that applying research evidence is not straightforward. Clinical practice guidelines and health technology assessments have emerged as a source of support. Clinical practice guidelines are becoming increasingly popular, *i.e.*, statements that include recommendations intended to optimize patient care that should be informed by a systematic review of evidence and an assessment of the benefits and harms of alternative care options [13,14].

Guidelines are considered a convenient way of packaging evidence and present recommendations to healthcare decision makers. Nevertheless, decisions should be influenced not only by the best estimates of the expected benefits and harms of a therapy or intervention but also by other factors. These include the confidence in these estimates (quality of the evidence), patient values, preferences, and for policy makers in particular, resource use, feasibility, and equity might also be relevant. Guideline developers have been inconsistent in how they rate quality of evidence and grade strength of recommendations, despite the critical role of these processes in guideline production [15]. As a result, guideline users face challenges in understanding guidelines’ messages and question their rigour, limiting their trustworthiness. Bridging the gap between clinical research and everyday healthcare practice requires more effective communication strategies.

### The GRADE system

The GRADE Working Group—a widely representative international group of guideline developers, health professionals, epidemiologists and statisticians—has spent over a decade developing an approach towards assessing and communicating the quality of evidence and the strength of recommendations (http://www.gradeworkinggroup.org). The GRADE approach is now well-established and is widely used internationally [16-20].

The GRADE Working Group has focused on a system for structuring judgements about the quality of evidence and characterising the strength of recommendations. In addition, the Working Group has developed and evaluated ways of presenting concise summaries of the findings of systematic reviews (as the basis for recommendations or decisions) to health professionals, and has contributed to ways of presenting this information to guideline developers, policymakers and patients [21-28]. The group has also addressed considerations for applying the GRADE system to recommendations about diagnostic tests and health system policies [23,24,29]. This work has been essential but does not address issues around how best to present and deliver GRADE recommendations to health professionals, policymakers, patients, and others. The Developing and Evaluating Communication Strategies to Support Informed Decisions and Practice Based on Evidence (DECIDE) project aims to build on this work by developing and evaluating ways of effectively communicating evidence-based recommendations to different target groups (see Table 1 for the list of DECIDE partners).

**Table 1:** DECIDE Partners

**Partner number**	**Participant organisation name**	**Participant short name**	**Country**
1	University of Dundee	UNIVDUN	United Kingdom
2	Norwegian Knowledge Centre for the Health Services	KS	Norway
3	Biomedical Research Institute (IIB-Sant Pau)	SANTPAU	Spain
4	Lazio Regional Health Service, Department of Epidemiology	ASL RME.DE	Italy
5	University of Amsterdam	UA	Netherlands
6	World Health Organisation	WHO	International
7	University Hospital, Freiburg	UHF	Germany
8	National Institute for Health and Clinical Excellence	NICE	United Kingdom
9	Scottish Intercollegiate Guidelines Network	SIGN	United Kingdom
10	Finnish Medical Society Duodecim	FMS	Finland

### Aims and objectives

DECIDE’s objectives are to develop and evaluate strategies for the targeted communication of evidence-based recommendations to the key stakeholders who determine what happens in healthcare. We will develop and evaluate strategies for effectively and efficiently communicating and supporting the uptake of evidence-based recommendations to: healthcare professionals; policymakers and managers; and patients and the general public.

In addition to addressing recommendations about prevention, treatment, and rehabilitation, we will develop strategies for recommendations about diagnostic tests (targeted mainly at healthcare professionals but could be others) and health system policies (targeted mainly at policymakers).

To ensure wide communication of DECIDE’s results we will: develop a tool kit for preparing and effectively communicating evidence-based recommendations; develop a database of evidence profiles (see ‘Development of a database of evidence profiles’ below); and host a European conference on promoting the optimal development and communication of evidence based recommendations.

DECIDE as originally envisaged has not been placed in a particular knowledge transfer or implementation framework. Instead, it aims to provide empirical support for a range of communication strategies, particularly how research evidence is presented to users to optimize access and use of the information contained within guidelines. A recent framework for guideline implementability [30] does, however, support many of the approaches taken by DECIDE, for example, tailoring guidelines for different types of user, grading evidence, presenting research evidence in a range of formats, considering how recommendations are presented, improved navigation, presenting information on patients’ and others’ preferences and values, information on costs, and providing information to support shared decisions between patients and clinicians. So, while DECIDE, is not explicitly placed within any particular knowledge transfer framework, it addresses key features of guidelines that are consistent with recent efforts to develop a guideline implementation framework.

DECIDE started on 1 January 2011 and will run for five years (http://www.decide-collaboration.eu/).

## Methods

The DECIDE project has organized its empirical work around five work packages (WPs), each aimed at a different stakeholder group or type of recommendation: health professionals (WP1); policymakers and managers (WP2); public, patients and carers (WP3); diagnostic tests (WP4); and health system policies (WP5).

There are three other work packages in DECIDE: WP6 will develop a toolkit for preparing and disseminating evidence-based recommendations (see below); WP7 will support communication; and WP8 is project management.

All work packages involve work in all DECIDE partner countries, namely Finland, Germany, Italy, the Netherlands, Norway, Spain, and the United Kingdom (UK) (see Table 1). Through collaboration with our World Health Organisation (WHO) partner, the GRADE Working Group, and the Guidelines International Network (G-I-N), this work will extend to other European countries, North America, and elsewhere.

Although work packages may develop different communication strategies, each focused on the needs of the particular stakeholder group, each of work packages 1 – 5 will use a similar approach to developing its strategies. This will comprise three phases:

### Phase one: strategy development and user testing

This work will collect user feedback from people in each of the targeted groups (*e.g.*, health professionals) through user testing, plus feedback from key stakeholders, for example, people who author guidelines. We will analyse feedback, define problem areas, and revise the strategies in brainstorming workshops. Additionally, we will survey user perceptions of a variety of current guidelines and their preferences regarding guideline content and presentation.

### Phase two: evaluating the communication strategies

The strategies coming from phase one will be evaluated, generally in randomized trials. The evaluations will be tailored to the different groups being targeted (*i.e.*, health professionals, policymakers and patients) and the different types of interventions (treatment, diagnostic and health systems).

### Phase three: testing our strategies with real guidelines

The strategies developed in phase two will be tested by using them prospectively in real guidelines prepared by consortium partners and collaborators. We will evaluate the impacts of these strategies on outcomes such as knowledge, attitudes and self-reported behaviour using surveys and interviews.

DECIDE will also develop a toolkit for guideline developers to support them in developing and communicating evidence-based recommendations using the DECIDE strategies, which will include a database of evidence profiles (see ‘Strategies for collaboration among European guideline developers and health technology assessment agencies in Europe (WP6)’ below).

**Figure 1:**
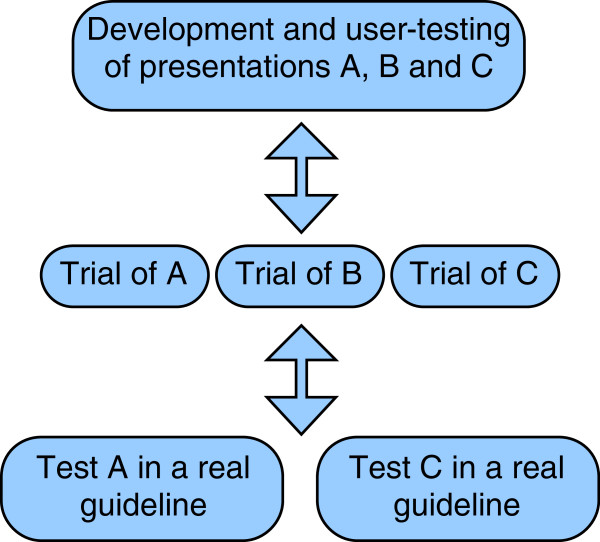
Simplified flow diagram of how the three phases of DECIDE will work.

**Figure 2:**
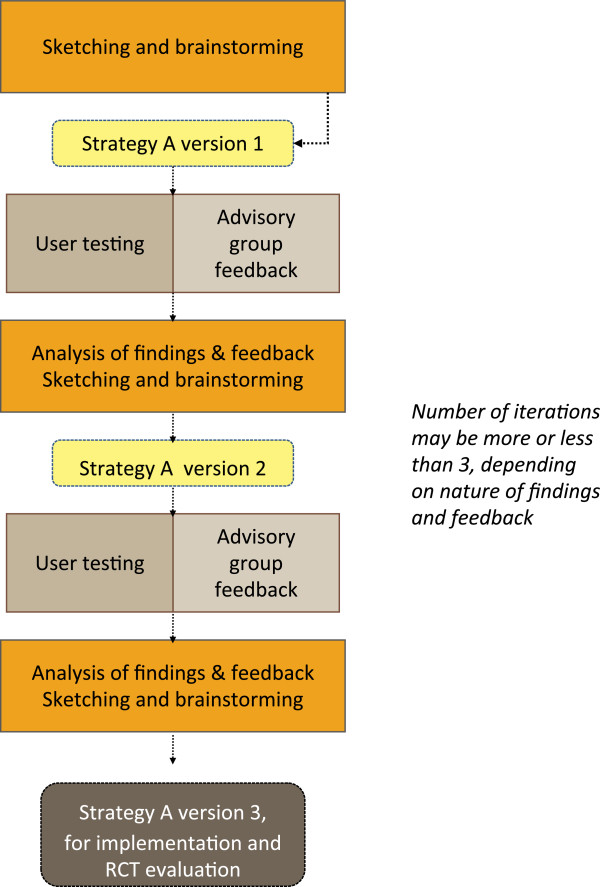
The iterative nature of the DECIDE strategy development process.

A simplified flow diagram of how the three phases of DECIDE will work in practice is given in Figure 1. The diagram is illustrative; we do not know at this stage how many strategies will go into phase two before the completion of phase one. The components of phase one will be staggered, rather than parallel. Moreover, the process will be iterative within phases (see Figure 2) and between phases in that we anticipate returning to, for example, phase one in light of what we learn in phase two.

Finally, ethics approval will be sought for each of DECIDE’s studies separately according to national regulations.

### Phase one: strategy development and user testing

Our initial development of communication strategies to effectively communicate evidence-based recommendations to stakeholders will be based on the work of the GRADE Working Group, Cochrane Collaboration Summary of Findings [21,22,31,32], SUPPORT summaries for policymakers and managers (http://www.support-collaboration.org/) [23,24], and plain language summaries for patients and the general public [25-27]. From this starting point, we will use multiple methods to develop templates for presenting evidence-based recommendations, supporting material, and communication strategies to health professionals, policymakers, or the public:

1. Brainstorming workshops (*e.g.*, with DECIDE partners) to generate ideas and solutions to problems uncovered through feedback and testing.

2. Literature reviews to inform development of communication strategies.

3. Stakeholder feedback (*e.g.*, from health professionals, policymakers, guideline authors) to inform development and revisions from diverse perspectives.

4. User testing (*e.g.*, with members of the public). To inform revisions from a user perspective.

5. Surveys of users to explore their understanding of guidelines and perceptions of a variety of current guidelines and their preferences regarding guideline content and presentation.

### Brainstorming workshops

At a minimum, brainstorming workshops with DECIDE partners working on each work package will take place at each annual project meeting and then after each round of stakeholder group feedback and user testing. We will apply principles from our professional perspectives including clinical epidemiology and information design, as well as diverse clinical backgrounds and experience developing and using guidelines. We will identify problems with these examples and ways in which communication could be improved by changes in presentation of the tables and recommendations, accompanying materials, and supportive strategies.

### Literature reviews

If relevant reviews do not already exist, then work packages will do one or more reviews to collate what is known about guideline dissemination methods for particular target groups. WP3, for example, will review the literature covering the evidence around patient (lay, public, citizen, consumer) beliefs, feelings, awareness, understanding and knowledge, expectation and perception of healthcare guidelines, and to collate knowledge on methods of communicating guideline recommendations to this audience. WP4 will review the grading systems used to grade evidence on diagnostic tests, which will inform work on how this might be improved and how the results of the grading might best be presented. WP1 will do two rapid systematic reviews, one looking at how guidelines and secondary resources (*e.g.*, UptoDate) present recommendations and evidence summaries, and a second looking for what has been studied about the different aspects around presentation of recommendations and evidence summaries. The results of all these reviews will inform DECIDE’s work, as well as advance knowledge around presenting guideline information to a wide range of stakeholders.

### Stakeholder feedback

DECIDE’s communication strategies will be informed by wide consultation with stakeholder groups. Each of work packages 1 – 6 will have an advisory group, comprising stakeholders relevant to the work package such as health professionals from diverse settings, guideline developers, policymakers, members of the public, patients and patient representatives, journalists, and researchers with expertise in clinical epidemiology and statistics, implementation science, communication, and psychology. Groups will be purposely selected to ensure a breadth of perspectives and will provide guidance on strategy, protocols, and specific approaches and tools throughout the project.

We will prepare summaries and recommendations for selected clinical topics using the strategies that are agreed on at the first brainstorming workshop. We will include topics that could be relevant to all partners (*e.g.*, depression, or cervical cancer screening). Less frequently, topics might be of interest to a single or just some participant countries. The summaries and recommendations we prepare will be used as the basis for gathering feedback on the format, content and delivery method of GRADE evidence-based recommendations and supporting materials in all of the participating countries. It is hard to predict what issues stakeholders will raise, but feedback could, for example, relate to the merits of quantitative versus qualitative presentation of information, the quality of information presented, or information missing from presentations.

We will consult stakeholders by email, encouraging them to collect feedback from their colleagues or constituencies when reporting back to us. In some cases, it may be more appropriate to use face-to-face discussions or focus groups. Our analysis will consider issues with a high level of agreement or disagreement, issues we had not previously considered, or issues considered to be of critical importance. We will consult the stakeholder groups as needed throughout the project.

### User testing

Participants from each partner country will take part in the user tests. The type of participant will vary depending on the work package (*e.g.*, health professionals for WP1, policymakers for WP2) and the stage of the work (*e.g.*, WP3 may start work with members of the public, but also discuss communication strategies with health professionals as the work progresses). We will guide test participants through a series of questions to explore which parts of the recommendations and supporting materials cause them problems, probing to better understand the nature of these problems.

We will use methods that members of the consortium have used for similar work [21-27]. In brief, each user test will take approximately one hour. Generally, and with the participant or participants’ written permission, we will audio-record each test, and an observer will take notes. Using a semi-structured interview guide, we will explore both immediate first impressions as well as detailed descriptions. The interview guide will be designed to explore seven different facets of ‘user experience’ (as originally described in a model by Peter Morville, [33] and adjusted after evaluation in a set of studies [34]): findability, usefulness, usability, understandability, credibility, desirability, and affiliation). The eighth facet from this model, accessibility, will not be addressed if user testing is done on paper, but will be explored if user testing is done with electronic presentations. Follow-up questions will cover overall impressions and suggestions for improvement.

We will review all of the notes and transcriptions, looking primarily for barriers and facilitators related to correct interpretation, ease of use, and favourable reception. We will trace findings back to specific elements or characteristics of the materials that appeared to facilitate or hinder problems. We will rate findings in three categories according to the severity of the problem for the user: high (causes incorrect interpretation, critical errors or high degree of uncertainty or dissatisfaction); medium (causes much frustration or unnecessarily slow use); or low (minor or cosmetic problems). We will also register nice-to-haves (things users explicitly liked) and suggestions for improvement.

### Surveys

We will survey representative samples of our targeted users (*e.g.*, the public or policymakers) regarding their understanding of guidelines, or their perceptions of a variety of current guidelines on the same topic (*e.g.*, the G-I-N, with which our consortium collaborates, lists 23 guidelines on atrial fibrillation, 103 on stroke, 26 on falls in the elderly, and 41 on prevention and intervention in influenza) and their preferences regarding guideline content, presentation of recommendations and current strategies used to disseminate research evidence. The surveys may also be used to evaluate alternative potential presentations formats. For example, we might ask health professionals about which icons they prefer in electronic guideline presentations.

The results of phase one will be collated and lead to a number of potential DECIDE strategies that will go on to phase two (evaluation).

### Phase two: evaluating the communication strategies

The various DECIDE strategies coming from phase one will be rigorously evaluated in partner countries, generally through a randomized trial. These strategies are likely to include the way documents are worded, how numerical information is summarized, graphical presentations, layering of information and electronic versus paper presentations. The strategies may differ between work packages although common elements are likely (*e.g*., common explanations of terminology). The objectives of the evaluations are to assess the impact of the various DECIDE strategies on intended behaviour and attitudes, as well as correct comprehension of key information and general satisfaction.

We will use a range of methods to run the evaluations, depending on the stakeholder group being targeted. For health professionals, we aim to conduct trials at health professional continuing education meetings such as rounds, workshops, and conferences in each of the DECIDE partner countries. Where possible, we will also target guideline users when they consult guidelines online in their clinical practice, when they visit websites of guideline developers, or through their scientific societies. Only practicing health professionals will be included. Members of the GRADE Working Group and people who have participated in developing the DECIDE strategies will be excluded. For policymakers, we will organize meetings with policymakers in each of the DECIDE partner countries. We will include policymakers and managers responsible for making or advising decisions about coverage, funding, and implementation of clinical practice guidelines or quality of care. Trials involving the public will involve mailing materials to individuals by post, or email, using a variety of methods, including those from earlier work presenting participants with scenarios and then measuring attitudes and intended behaviour [35]. Participants will be identified in a variety of ways, including through health databases, registers of individuals interested in taking part in research, and public and patient groups.

In each trial, we will randomize participants to receive information and clinical recommendations using one of the proposed DECIDE strategies for that work package or an alternative, which might be a conventional strategy (*e.g.*, a recommendation taken verbatim from one or more existing guidelines), or another DECIDE strategy. Discussions with stakeholder groups will provide us with estimates of the minimal important difference in outcomes upon which to base sample size calculations. The trial questionnaires will include multiple-choice questions measuring correct comprehension of the specific recommendation and supporting material, attitudes, hypothetical or intended behaviour, and satisfaction. The measurement of attitude and intention will be informed by appropriate behavioural theory. Participants’ responses will be anonymous. Where the trial is run as a face-to-face meeting, structured discussions will be carried out at the end of the meeting, and we will interview selected participants to explore strengths and weaknesses of the strategies, reasons for their success or failure, and ways of improving them.

These trials will tell us if one or more of the DECIDE strategies supports increased understanding, user satisfaction, and/or behaviours (*e.g.*, intention to prescribe a particular drug supported by a recommendation). For example, we may find that one DECIDE communication strategy allows participants to both find and understand the balance of benefits and harms much better than a second DECIDE strategy and the existing guideline presentation. Another trial might have clinicians reviewing a number of clinical scenarios after first having been presented with guideline recommendations presented in one of two formats. For example, measurement of intention to refer patients in the scenarios to screening may be more closely aligned with the guideline recommendations with one format than another. If participants are not too heterogeneous it may be possible to pool results from several trials. Interview data will help to explain and interpret the results of the quantitative analysis. The trials will allow us to sift out the least promising strategies prior to moving to phase three.

### Phase three: testing our strategies with real guidelines

The most promising DECIDE strategies for each work package will be revised as needed based on the results of phase two. The revised strategies will then be tested by using them with real clinical practice guidelines prepared by DECIDE partners and other collaborators. Other developers linked to the GRADE Working Group will also be involved (see http://www.gradeworkinggroup.org/society/index.htm). These guideline developers will use the strategies for communicating evidence-based recommendations and the supporting material generated during phases one and two with their new or updated guidelines. We will test our strategies for optimal dissemination of recommendations through different designs such as interrupted time series, surveys and randomized trials of alternative presentation formats.

We will evaluate the impacts of these strategies on knowledge, attitudes, and self-reported behaviour of guideline users using electronic means (email or web-based systems) before and after the DECIDE strategies are used to present recommendations supplemented by interviews (after communication of the recommendations).

### Strategies for collaboration among European guideline developers and health technology assessment agencies in Europe (WP6)

We will develop a toolkit for preparing and communicating evidence-based recommendations. Development of the toolkit will be based on the GRADE profiler (GRADEpro) software developed by members of the GRADE Working Group and used by a wide variety of organisations, including the DECIDE partners. GRADEpro is an application for developing, managing, and sharing evidence profiles and Summary Of Findings Tables and making recommendations. This tool is intended for authors of systematic reviews, guideline developers, and those requiring summaries of the best available evidence for recommendations about particular courses of action in healthcare. An evidence table is a key tool in the presentation of evidence and the corresponding results, and it displays the information about all outcomes for a given healthcare question in tabular format, information that should include a systematic review of the best available evidence. Health professionals, patients and the public, guideline developers, and policymakers should be able to access succinct, explicit, and structured evidence summaries to inform their decisions. While an unambiguous healthcare question is key to evidence summaries, the requirements for specific users may differ in content and detail. The Decision Table, another table developed by GRADEpro, includes the rationale for making recommendations based on the underlying quality of evidence, the balance between benefits and harms, values and preferences, and resource considerations. This table is used to lay out judgments that guideline developers make when they develop recommendations [36].

As part of the DECIDE project, we will further develop GRADEpro to incorporate presentations (including recommendations, text, tables and, if appropriate, figures) developed in the five empirical work packages described above (*i.e.*, WPs 1 to 5) to support communication to health professionals, policymakers, and members of the public, as appropriate. The software will support materials in several languages including Dutch, English, French, German, Italian and Spanish. We will encourage GRADE members from other European countries to provide a translation for their country’s language. The revised and expanded software will be tested by all of the DECIDE partners and by other members of the GRADE Working Group and users, including Cochrane Collaboration review authors. The final product will be an interactive, browser-based guideline development tool.

### Development of a database of evidence profiles

To facilitate collaboration across European guideline developers, we will develop a database of evidence profiles prepared using GRADEpro and the strategies developed by DECIDE. An evidence profile consists of two components: an assessment of the quality of evidence and a summary of the findings [37]. This work will build on a pilot database developed by the GRADE Working Group (http://www.gradeprofiles.org/). We will establish an advisory group with representatives from producers of guidelines and systematic reviews to obtain input into key decisions, including quality control, inclusion of metadata, and access to the database. In addition to organisations that are partners in DECIDE, we will invite representatives from the Cochrane Collaboration, the G-I-N, and the International Network of Agencies for Health Technology Assessment (INAHTA) to join the advisory group. The role of the advisory group will be to help ensure that the database is optimally designed to minimize unnecessary duplication of efforts and maximize access to evidence profiles by guideline developers and HTA agencies in Europe.

The profile database will accommodate the changing and evolving nature of the grading methodology over time (such as empirical evidence from DECIDE). This makes storing static documents (such as pdf files of finished evidence profiles) less appealing. Instead, the database will store the individual data points, *e.g.*, effect sizes, and the evidence profile will be re-created on demand. This allows for flexibility in providing different profile presentations that can be utilized for targeted user testing in randomized trials and allows creating different output formats, such as pdf files, rich text formats, or in graphical form. In addition, the original data set can be downloaded at any time for reuse and for easy updating at a later time point or by other authors. GRADE profiles will be conceptually stored in three separate components: the profile identifiers (*e.g.*, profile name), patient or population-important outcomes (including quality of evidence rating and estimate of effect), as well as annotations (footnotes, which provides the rational for the evidence rating and additional explanations).

Other tools and resources may also be developed. For example, documents providing guidance to guideline developers about how best to present information to the public or policymakers.

### Preliminary results

**Figure 3:**
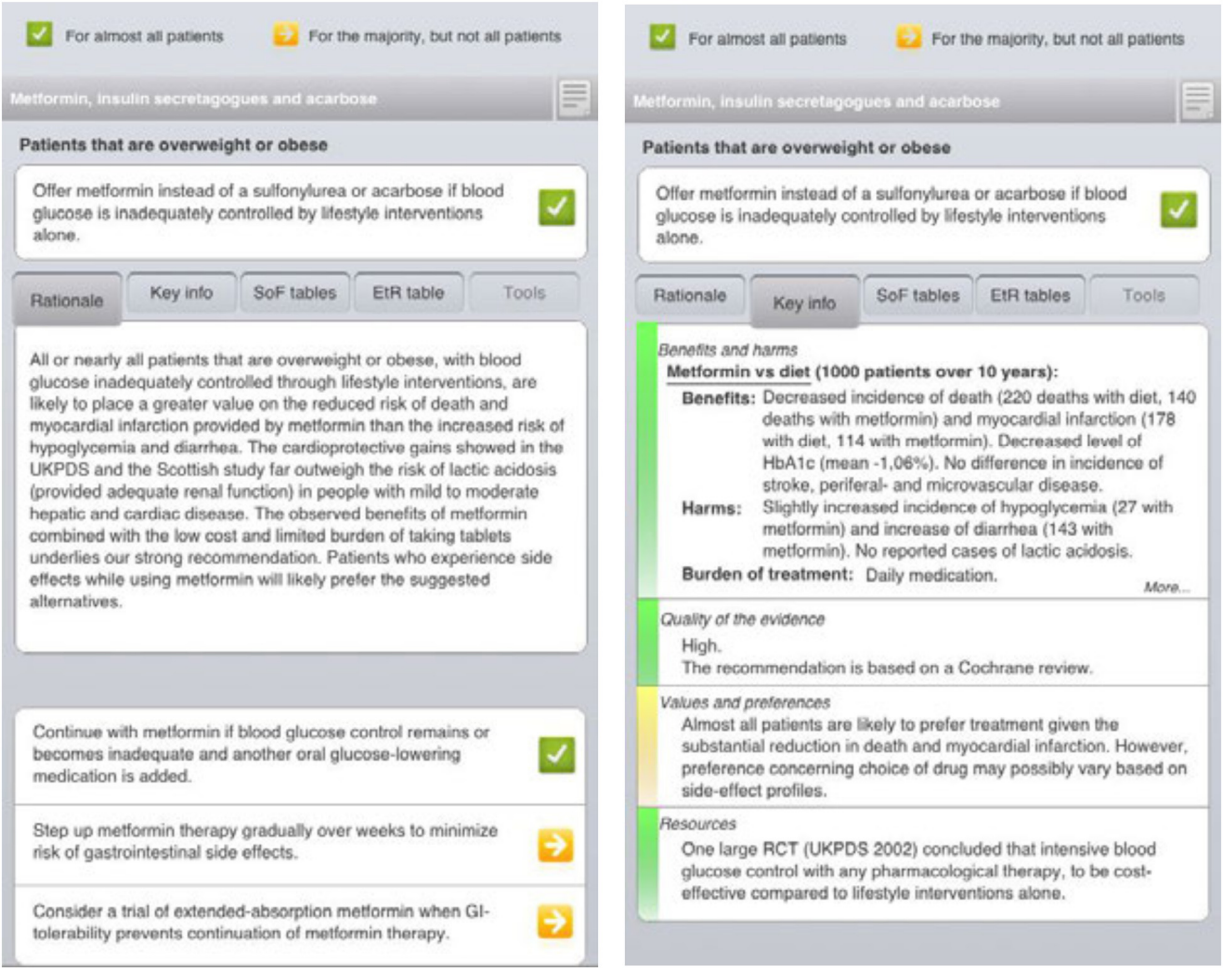
Prototype of a DECIDE communication strategy for health professionals.

Table 2 provides an overview of the strategies currently being developed and user tested for work packages 1 to 5. The preliminary results are available at the DECIDE webpage (http://www.decide-collaboration.eu/work-packages-strategies). WP1’ user-testing of presentation formats has so far resulted in an electronic multilayered guideline format to be made available in outputs for multiple platforms (*e.g.*, for websites, smartphone apps, and electronic health records). Figure 3 gives an example of this multilayered presentation format with what we call the guideline top layer, which presents the minimum amount of information that a health professional would need to understand and act on a recommendation. The top layer presentation is being implemented in an adaptation of a national antithrombotic guideline in Norway [38]. This guideline will be presented in an electronic multilayered guideline on the web and in smartphone applications.

**Table 2:** Strategies being developed by DECIDE

	**WP1 clinicians**	**WP3 consumers**	**WP4 diagnostic tests**	**WP2 coverage decisions**	**WP5 health system decisions**
**Presentation of evidence and recommendations**	Top Layer presentation				
	Explanations of key concepts				
	Interactive Summary of Findings tables/ videos				
**Frameworks for going from evidence to recommendations**	Evidence to recommendation frameworks			* See note	Evidence to recommendation framework
				Costing frameworks	
**Decision support**	Decision aids		Decision aids and Evidence to decision frameworks	Evidence to decision frameworks	
**Communication strategies**	Point of care applications Multilayered guidelines in electronically structured outputs	Point of care applications and Guidance and tools for guideline producers	Adaptation of point of care applications and Guidance and tools for guideline producers		

* Recommendations for coverage decisions are not common. Typically these decisions are made by responsible groups in each jurisdiction or organisation, although sometimes technical support staff will recommend a decision that is then considered by those responsible for making the decision.

WP1 has through this research identified the need to develop the presentation formats in parallel with development of the Guideline Development Tool to facilitate the authoring of guideline content and creation of the various electronic outputs. This will require specific functionality in the Guideline Development Tool to be developed in WP6. Work packages 1, 3, 4, and 5 are developing frameworks for going from evidence to recommendations as a means of supporting recommendations that are well informed by evidence. These frameworks build on the previous work of the Decision Table. WP2 (policymakers) will focus specifically on coverage decisions, for which recommendations are uncommon. WP1 is also developing an Evidence To Recommendation Table for healthcare professionals. This table aims to be a friendlier Summary of Findings Table that includes other features as values and preferences and resource use.

The Evidence to Recommendation Frameworks (see Additional files 1, 2 and 3 for examples) aim to provide the basis for communicating to users the justification for a recommendation and the basis for decision support tools, which are being developed for all five work packages. For individual patient decisions, these tools will be decision aids or recommendation presentations that can be derived directly from Summary of Findings Tables and the Evidence To Recommendation Frameworks. Those responsible for policy decisions will be assisted by Evidence to Decision Frameworks, decision support tools that will assist decision makers to transparently make judgements, and eventually decisions, informed by available evidence. Clear definitions of the criteria used in the frameworks will be provided in order to increase transparency of the process.

Work packages 2 and 5 are in addition developing and testing frameworks for considering costs in relation to coverage decisions and health system decisions respectively.

We are additionally developing and testing explanations of key concepts and interactive Summary of Findings Tables that will be adapted for use across all five work packages and their final strategies (*e.g.*, Evidence to Recommendations Frameworks). The explanations will include brief explanations of concepts such as ‘quality of evidence’ or ‘confidence intervals’ that can, for example, be used as cursor-over help in guidelines, or Summary of Findings Tables. In addition, we will develop longer explanations using videos, interactive applications, or other presentations to facilitate understanding. These can be provided as help using hypertext links in, for example, online guidelines, as resources or a help file on guideline producers’ websites or in resources, such as the Cochrane Library, as an open access online resource, or as an introduction to a group making recommendations or decisions. The objective of this interactive work is to improve understanding and use of evidence of the effects of healthcare interventions by developing products (*e.g.*, interactive Summary of Findings Tables) that allows producers to tailor a presentation to different target audiences and for users to interact with the presentation.

The Guideline Development Tool (http://www.guidelinedevelopment.org) will build on the features of GRADEpro and include:

1. Learning modules about a guideline development.

2. Export of evidence syntheses to a database of evidence profiles.

3. Support for communication, polling, managing conflict of interest, voting, and other forms of interaction among multiple stakeholders providing input (*e.g.*, guideline panel members).

4. Ergonomic text editor with essential functions including text formatting, tables, inserting images, providing footnotes and explanations, tracking changes, and inserting references from an integrated reference manager.

5. Ability to create templates to produce various types of electronic and printable documents based on the information collected at each step of the process.

6. Interactive Summary of Findings Tables.

The full list of functionality, as well as the current list of learning modules being developed for the Guideline Development Tool is available in Additional file 4.

Additional communication strategies for clinicians will include point of care applications to support the provision of clinical recommendations linked to medical records, as parts of clinical decision support systems, and on smartphones or tablets. For patients, these will include access to decision aids at the point of care, to be used in consultations with their clinicians, and tools to assist guideline developers in developing versions of guidelines that are easily accessible to targeted patients.

## Discussion

DECIDE will directly address how information about healthcare interventions are created, packaged, transmitted, and interpreted among a variety of important stakeholder groups including healthcare professionals, healthcare managers, policymakers, and patients.

DECIDE will build on the substantial experience and knowledge of the GRADE Working Group. The GRADE Working Group has so far focused on a system for structuring judgements about the quality of evidence and strength of recommendations and there is an emerging consensus around this approach [16,18,20]. In addition, the Working Group has developed and evaluated ways of presenting concise summaries of the findings of systematic reviews (as the basis for recommendations or decisions) to health professionals, and has contributed to ways of presenting this information to guideline developers, policymakers, and patients [21-25,28]. The Group has also addressed considerations for applying the GRADE system to recommendations about diagnostic tests and health system policies [23,24,28]. This work has been essential but does not address issues around how best to package and deliver GRADE recommendations to health professionals, policymakers, patients, and others. In fact, little research has been done in this field [12,39,40].

Based on this work DECIDE will develop and evaluate ways of effectively communicating and supporting the uptake of evidence-based recommendations (and the basis for such recommendations). This work will advance the state-of-the-art by taking the successful GRADE system and providing new research data on the most effective ways of using GRADE to develop and disseminate research evidence to key clinical practice stakeholders across different countries, health systems, and settings in Europe. It will provide new information on how to tailor global research evidence to local needs. Moreover, DECIDE is also a partner in a research programme called MAGIC (Making GRADE the Irresistible Choice, http://www.magicproject.org/), which develops methodology and technology to integrate guidelines in the electronic medical record (as clinical decision support systems) and to facilitate local adaptation and dynamic updating of healthcare recommendations.

It is likely that the way in which recommendations are formulated needs to be adapted to the specific characteristics of a guideline. Providing empirically-based information on how this might be done is what DECIDE aims to achieve. DECIDE will advance the state-of-the art by first collecting data on stakeholders’ opinions of alternative ways of presenting recommendations and then obtain empirical evidence of the utility of these strategies by running a series of randomized controlled trials in a range of European countries. The data from these trials will be used to inform the optimal presentation of recommendations in real clinical guidelines produced by the guideline developers in our consortium, as well as others through the GRADE Working Group. DECIDE’s rigorous assessment of the effectiveness of communication strategies will provide an empirical basis for better understanding of the factors that influence the effectiveness of communication strategies on the various actors in the healthcare sector. The impact of these strategies on knowledge, attitudes, and self-reported behaviour will then be evaluated. The impact will therefore be felt not only at a clinical level, but across diverse healthcare settings within European healthcare systems and elsewhere.

## Competing interests

The authors declare that they have no competing interests.

## Authors’ contributions

All authors contributed to the development of the study. ST, PA and ADO wrote the first draft of the paper from the original grant proposal, with all authors commenting on it and subsequent drafts. All authors approved the final version.

## Members of the DECIDE consortium

University of Dundee, UK: Claire Hartley, Kirsty Loudon, William Slater, Neil Stewart, Shaun Treweek; Norwegian Knowledge Centre for the Health Services, Norway: Linn Brandt, Signe Flottorp, Claire Glenton, Annette Kristiansen, Simon Lewin, Jenny Moberg, Angela Morelli, Andy Oxman, Sarah Rosenbaum, Per Olav Vandvik, Jan Ødgaard-Jensen; Biomedical Research Institute (IIB-Sant Pau), Spain: Pablo Alonso-Coello, Laura Martinez-García, David Rigau, Ivan Solà, Andrea Juliana Sanabria. Lazio Regional Health Service, Italy: Laura Amato, Massimo Brunetti, Marina Davoli, Nicola Magrini, Elena Parmelli, Francesco Nonino, Rossana De Palma, Donato Papini, Silvia Pregno, Carlo Saitto; University of Amsterdam, the Netherlands: Patrick Bossuyt, Gowri Gopalakrishna, Miranda Langendam, Mariska Leeflang, Rob Scholten; World Health Organisation: Metin Gülmezoglu, Govin Permanand, Krisantha Weerasuriyak; University Hospital Freiburg, Germany: Gerd Antes, Jörg Meerpohl, Holger Schünemann; National Institute for Health and Clinical Excellence, UK: Phil Alderson, Emma McFarlane, Judith Thornton; Scottish Intercollegiate Guidelines Network, UK: Margaret Callaghan, Karen Graham, Robin Harbour, Karen Ritchie, Duncan Service; Finnish Medical Society Duodecim, Finland: Ilkka Kunnamo, Helena Liira; McMaster University, Canada: Gordon Guyatt, Reem Mustafa, Ignacio Neumann, Nancy Santesso, Fred Spencer; Case Western Reserve University, USA: Yngve Falck-Ytter; University of Chile, Chile: Romina Brignardello-Petersen, Alonso Carrasco-Labra; University Hospital Basel, Switzerland: Regina Kunz; University of Buffalo, USA: Elie Akl.

## Supplementary Material

Additional file 1 Example framework from WP1. Click here for file

Additional file 2 Example framework from WP2. Click here for file

Additional file 3 Example framework from WP5. Click here for file

Additional file 4 The Guideline Development Tool. Click here for file
