# Supplementary material for: Developing and evaluating communication strategies to support informed decisions and practice based on evidence (DECIDE): protocol and preliminary results
Source: Implement Sci. 2013 Jan 9;8:6. doi: 10.1186/1748-5908-8-6 (PMC3553065; doi:10.1186/1748-5908-8-6)
Supplement: Additional file 1 — Example framework from WP1. [file 1748-5908-8-6-S1.pdf]

# Should warfarin vs. aspirin therapy be used for patients with atrial fibrillation and intermediate risk of stroke (CHADS2=1)?

**Population**: patients at intermediate risk of stroke (CHADS2 score of 1)
**Intervention**: warfarin
**Comparison**: aspirin
**Setting**: outpatients

| | | JUDGEMENT | DETAILS OF JUDGEMENT | EVIDENCE/EXPLANATION |
|---|---|---|---|---|
| QUALITY | **What is the overall quality of evidence?** | ☐ High<br>☑ Moderate<br>☐ Low<br>☐ Very low | See table Quality below | • Imprecision was a reason for downgrading for nonfatal major bleeds, death and systemic embolisms |
| BENEFITS & HARMS | **What is the balance between benefits and risks/ burden?** | *Group:*<br>☐ Benefits outweigh harms/ burden<br>☑ Benefits slightly outweigh harms/ burden<br>☐ Benefits and harms/ burden are balanced<br>☐ Harms/ burden slightly outweigh benefits<br>☐ Harms/ burden outweigh benefits | See table Benefits & Harms below | See evidence table below |
| VALUES AND PREFERENCES | **What are the patient's values and preferences, and what certainty do we have about them?** | ☐ Little unceirtany and similar values<br>☑ Some uncertainty or some variation<br>☐ Significant uncertainty or large variation | See table Values and Preferences below | • We are moderately confident that patients will place the greatest value on avoiding strokes. Our best estimate from a systematic review is that 1 stroke equals 1 major bleed. There is wide variability in these values and preferences. Patients averse to taking oral anticoagulants for their potential of bleeding may be reluctant to use long-term warfarin therapy. |

**Quality – Details of judgement**

| *Critical Outcomes:* | *High* | *Moderate* | *Low* | *Very low* |
|---|---|---|---|---|
| 1. Death | ☐ | ☑ | ☐ | ☐ |
| 2. Non fatal strokes | ☑ | ☐ | ☐ | ☐ |
| 3. Non fatal extracraneal major bleeds | ☐ | ☑ | ☐ | ☐ |
| 4. Systemic embolism | ☑ | ☐ | ☐ | ☐ |
| 5. Burden of treatment | ☑ | ☐ | ☐ | ☐ |

**Benefits & Harms – Details of judgement**

| *Critical Outcomes:* | *Large/Modest benefit* | *Small benefit* | *No effect* | *Small harm/ burden* | *Modest/Large harm/ burden* |
|---|---|---|---|---|---|
| 1. Death | ☐ | ☐ | ☑ | ☐ | ☐ |
| 2. Non fatal strokes | ☑ | ☐ | ☐ | ☐ | ☐ |
| 3. Non fatal extracraneal major bleeding | ☐ | ☐ | ☐ | ☑ | ☐ |
| 4. Systemic embolism | ☐ | ☐ | ☑ | ☐ | ☐ |
| 5. Burden of treatment | ☐ | ☐ | ☐ | ☑ | ☐ |

**Benefits & Harms – Evidence/Explanation**

| Outcome | Absolute estimates (95% CI) Time frame: 1 year | | | Relative estimates (95% CI) | Patients (studies) |
|---|---|---|---|---|---|
| | Intervention | Control | Difference (per 1000) (95% CI) | | |
| Death | 46/1000 | 47/1000 | 1 fewer (from 7 fewer to 6 more) | 0.97 (0.85-1.12) | 5855 (10) |
| Non Fatal strokes | 8/1000 | 17/1000 | 9 fewer (from 5 fewer to 11 fewer) | 0.48 (0.33-0.70) | 6526 (11) |
| Non fatal major bleeding | 12/1000 | 8/1000 | 3 more (from 1 fewer to 10 more) | 1.42 (0.49-2.29) | 6526 (11) |
| Systemic embolism | 2/1000 | 3/1000 | 1 fewer (from 2 fewer to 2 more) | 0.81 (0.40-1.64) | 6256 (11) |
| Burden of treatment | The use of warfarin compared to aspirin increases the burden of treatment, due to the need of more frequent controls | | | | |

**Values and Preferences – Details of judgement**

| | *Agree* | *Somewhat agree* | *Uncertain* | *Somewhat disagree* | *Disagree* |
|---|---|---|---|---|---|
| High confidence in the tipical values | ☐ | ☐ | ☐ | ☑ | ☐ |
| Values and preferences likely similar | ☑ | ☐ | ☐ | ☐ | ☐ |

| | | | | |
|---|---|---|---|---|
| RESOURCES | **Is the incremental cost (or resource use) small relative to the benefits?** | ☐ Cost is very small relative to the benefits<br>☑ Cost is small relative to the benefits<br>☐ Cost is borderline relative to the benefits<br>☐ Cost is high relative to the benefits<br>☐ Cost is very high relative to the benefits | *Agree / Somewhat agree / Uncertain / Somewhat disagree / Disagree*<br>Costs are low: ☐ ☑ ☐ ☐ ☐<br>Benefits are important: ☐ ☑ ☐ ☐ ☐ | • Resources required seem to worth the net benefit.<br>• Both drugs are of low cost. Treatment with warfarin costs USD 5 more per patient per month[ζ].<br>• Treatment with warfarin needs tight monitoring controls, increasing the burden of treatment. |

| | | | | | | |
|---|---|---|---|---|---|---|
| **Balance of consequences** | Undesirable consequences *clearly outweigh* desirable consequences<br>☐ | Undesirable consequences *probably outweigh* desirable consequences<br>☐ | The balance between desirable and undesirable consequences is uncertain*<br>☐ | There is a balance between desirable and undesirable consequences*<br>☐ | **Desirable consequences *probably outweigh* undesirable consequences**<br>☑ | Desirable consequences *clearly outweigh* undesirable consequences<br>☐ |

| | | | | |
|---|---|---|---|---|
| **Recommendation** | *We recommend against the option*<br>☐ | *We suggest not using the option*<br>☐ | ***We suggest using the option***<br>☑ | *We recommend the option*<br>☐ |
| | **In patients at intermediate risk of stroke (CHADS2=1) we suggest using warfarin (over aspirin)** | | | |
| **Recommendation rationale** | The majority of patients place a higher value on avoiding a stroke than experiencing a bleeding and the inconvenience associated with warfarin. Given the absolute stroke reduction in stroke we suggest the use of warfarin (over aspirin) as most patients if informed would choose this treatment. However, this absolute stroke reduction is considered small for some patients, and many would not choose this treatment. | | | |

* In this situation no recommendation could be reasonable

ζ Hypothetical information
