# Supplementary material for: Developing and evaluating communication strategies to support informed decisions and practice based on evidence (DECIDE): protocol and preliminary results
Source: Implement Sci. 2013 Jan 9;8:6. doi: 10.1186/1748-5908-8-6 (PMC3553065; doi:10.1186/1748-5908-8-6)
Supplement: Additional file 2 — Example framework from WP2. [file 1748-5908-8-6-S2.pdf]

**Question**
Should Palivizumab be covered for immunoprophylaxis of respiratory syncytial virus (RSV) bronchiolitis in high-risk infants and young children?
**Background Information**
RSV causes outbreaks of respiratory tract infection in temperate areas, especially in the winter months. It can affect people of any age and is usually a mild, self-limiting illness. It is most serious in infants and young children, in whom it is the single most important cause of lower respiratory tract infection (LRTI). RSV infection can present with a wide range of severity from mild respiratory symptoms, to rhinitis and otitis media, through to bronchiolitis, trachea-bronchiolitis and pneumonia. The diagnosis of bronchiolitis is based only on clinical signs and symptoms.<br><br>The virus is spread by contaminated nasal secretions via respiratory droplets, so close contact with an infected individual or contaminated surface is required for transmission. RSV can persist for several hours on toys or other objects. Risk factors for RSV infection include crowding, low socioeconomic status, exposure to tobacco smoke and admission to hospital during the RSV season (late autumn to early spring). The children most at risk from severe disease if infected with RSV are infants under 6 weeks old or who have chronic lung disease (CLD), congenital heart disease (CHD) or immunodeficiency, and those born prematurely (at 35 weeks gestational age or before).<br><br>Approximately 4%-11% of infants and young children develop bronchiolitis during the first three years of life. Among those approximately 50% are infected by RSV (data from Italy).<br><br>The therapy for bronchiolitis due to RSV infection , both of moderate and severe degree, is based on ventilatory support and adequate hydration. The efficacy of ribavirin is uncertain. The prognosis is almost favorable.<br><br>Passive Prophylaxis with high-tittered human polyclonal RSV IVIg does not significantly reduce the incidence of RSV infections. However, monthly prophylaxis significantly reduced the severity of RSV infections in very young high-risk patients, reduce the hospitalization rates and significantly shorter hospital stays compared to well-matched control patients.<br><br>No vaccine are available.<br><br>Palivizumab is the only licensed product available for prevention of RSV lower respiratory tract disease in infants and children with CLD, with a history of preterm birth (<35 weeks' gestation), or with haemo-dynamically significant CHD. Palivizumab is a humanized<br><br>murine monoclonal anti-F glycoprotein immunoglobulin with neutralizing and fusion inhibitory activity against RSV and it is administered intramuscularly at a dose of 15 mg/kg once every 30 days.

Criteria	Evidence	Judgement
**Seriousness**<br>Is the condition severe (e.g. life threatening or disabling)?	Most of the infected children develop respiratory distress of low or moderate degree.<br>IN Italy the hospitalization for bronchiolitis ranges from 0,6% to 5%. Among those children about 30%-50% are infected with RSV (children both from high and low risk)<br>This variability is attributable to the different criteria for hospitalization and different tests used to diagnose the RSV infection.<br>The duration of hospitalization ranges from 5 to 6 days in Italy.<br>Mortality due to bronchiolitis is less than 1% in children infected with RSV without underlying illness (USA).<br>Mortality due to LRTI in those infected with RSV with heart and lung disease who are hospitalised is estimated to be around 3–5%.5 (USA).<br>On those basis about 6 deaths due to RSV infection are expected in the cohort of Italian newborns per year.	Yes ☐ Uncertain ☐ No ■
**Quality of evidence**<br>Can we be confident in the estimates of effect?	The efficacy of palivizumab has been evaluated in two different population (CHD and CLD) in two multicenter, placebo controlled, randomized clinical trials and the global quality of evidence is judge, following GRADE criteria, as LOW. The two trials have adequate concealment of allocation, double blind assessment, loss to follow-up clearly reported and and use of intention to treat analysis. Both studies measured as primary outcome the reduction in hospitalization rate as proxy of illness severity. They both present some flaws in directness dimension for the critical outcomes measured: the use of placebo in the control group instead of the prophylaxis with high-tittered human polyclonal RSV IVIg ; the use of unspecified type of tests for the detection of RSV infection. Both studies were underpowered to detect a difference in mortality between palivizumab and placebo group. Both studies were sponsored by the palivizumab manufacturer. The IMpact trial was also underpowered to detect a difference between subgroups and there is no evidence of a true underlying difference in effect size and it don't report relevant data for cystic fibrosis or immune deficiency subgroups.<br><br>The efficacy of palivizumab in preventing RSV infection was also evaluated in children with cystic fibrosis through a SR including one RCT with very important limitations. The global quality of evidence in this case turn out to be VERY LOW.	Yes ☐ Uncertain ■ No ☐

Benefits
Are the desirable effects large?

It is uncertain whether palivizumab reduce incidence of RSV hospitalization, days hospitalized, need of oxygen therapy, ICU hospitalization rate, need in mechanical ventilation and mortality when measured in the population as whole, both in premature children with or without CLD children, in children with CHD and in children with cystic fibrosis. It is also uncertain whether palivizumab reduce the incidence of the outcome above mentioned in subgroup of population analysed.

A=overall population
B=children of gestational age ≤ 24months old haemo-dynamically significant CHD and unoperated or partially corrected CHD
C=children without CLD
D=children with CLD
E=children of gestational age 32-35 weeks
F=children of gestational age <32 weeks
G=non-cyanotic children
H=cyanotic children

Outcome	Results	GRADE
Reduction in mortality	A, B: Inconclusive compared to placebo	⊕⊖⊖⊖ VERY LOW
Reduction in incidence of bronchiolitis	A, B: Not measured	NOT EVALUABLE
Reduction in long term complications	A, B: Not measured	NOT EVALUABLE
Reduction in ICU hospitalization rate	A, B: Inconclusive compared to placebo	⊕⊕⊕⊖ MODERATE
Reduction in need of mechanical ventilation	A, B: Inconclusive compared to placebo	⊕⊕⊕⊖ MODERATE
Reduction in days hospitalized for bronchiolitis	A: 42% reduction in risk compared with placebo (the difference in duration of hospitalization <1 day)<br><br>B: 56% reduction in risk compared with placebo (the difference in duration of hospitalization <1 day)	⊕⊕⊕⊖ MODERATE
Reduction in incidence of RSV hospitalization	A: 55% reduction in risk compared with placebo<br><br>B: 45% reduction in risk compared with placebo<br><br>C: 78% reduction in risk compared with placebo<br><br>D: 39% reduction in risk compared with placebo<br><br>E: 80% reduction in risk compared compared with placebo<br><br>F: 47% reduction in risk compared with placebo<br><br>G: 58% reduction in risk compared with placebo<br><br>H: Inconclusive compared with placebo	⊕⊕⊖⊖ LOW

The data reported above come from an HTA published in 2011.This document include RCTs affected by several methodological flaws that led their quality of evidence to be judge, using GRADE criteria, ranging from MODERATE to VERY LOW for the outcomes considered. That's why it is uncertain if the estimates of the effect are large.

Reduction in mortality in children with cystic fibrosis	Inconclusive compared to placebo	⊕⊖⊖⊖ VERY LOW
Reduction in incidence of bronchiolitis in children with cystic fibrosis	Not measured	NOT EVALUABLE
Reduction in long term complications in children with cystic fibrosis	Not measured	NOT EVALUABLE
Reduction in	Inconclusive compared to placebo	⊕⊖⊖⊖ VERY LOW

Yes	Uncertain	No
☐	■	☐

incidence of RSV hospitalization in children with cystic fibrosis		

These data come from a SR document published in 2010.This document include one RCT affected by several methodological flaws that leid his quality of evidence to be judge, using GRADE criteria, as VERY LOW for the outcomes considered. That' why it is uncertain if there is any benefit using palivizumab.

Adverse effects
Are the undesirable effects of the option small?

Outcome	Results	GRADE
Any adverse event	Inconclusive compared to placebo	⊕⊕⊖⊖ LOW

These data come from a HTA document published in 2011 and a SR published in 2010.The documents include RCTs affected by several methodological flaws that leid their quality of evidence to be judge, using GRADE criteria, as LOW. That's why it is uncertain if the undesirable effects are small. These data are related to all of the populations considered in evaluating the estimate of beneficial effects

Yes	Uncertain	No
☐	■	☐

Resource use (costs)
Are the costs low/affordable?

Average costs in children without CLD (£)			
	Palivizumab	No prophylaxis	Difference
Palivizumab	3437		
Drug administration	60		
Hospital	67	301	
Total cost (NHS)	3564	301	3263

Average costs in children with CLD (£)			
	Palivizumab	No prophylaxis	Difference
Palivizumab	3437		
Drug administration	60		
Hospital	293	475	
Total cost (NHS)	3790	475	3315

Average costs in children with acyanotic CHD (£)			
	Palivizumab	No prophylaxis	Difference
Palivizumab	3714		
Drug administration	60		
Hospital	359	647	
Total cost (NHS)	4132	847	3285

Average costs in children with cyanotic CHD (£)			
	Palivizumab	No prophylaxis	Difference
Palivizumab	3714		
Drug administration	60		
Hospital	402	567	
Total cost (NHS)	4176	567	3609

These data derive from a recent HTA (Wang 2011). Palivizumab was considered using a five doses scheme (see page 20).

Yes	Uncertain	No
☐	☐	■

Cost-effectiveness
Is the cost small relative to the net benefits?

A recent HTA (Wang 2011) calculated the cost effectiveness for different subgroups, but the authors recognized that there is a poor quality estimates. This data showed that prophylaxis with palivizumab does not represent good value for money based on the current UK incremental cost-effectiveness ratio threshold of £30,000/QALY when used unselectively in children without CLD/CHD or children with CLD or CHD. In summary, the cost effective subgroups (< £30,000/QALY) for children who had no CLD or CHD must contain at least two other risk factors apart from Gestational age and birth age. The cost-effective subgroups for children who had CLD or CHD do not necessarily need to have any other risk factors.

Yes	Uncertain	No
☐	■	☐

Feasibility
Is it feasible to avoid any administrative constraints and to ensure appropriate use?

Possible difficulties in professional acceptability due to the large variability in prescribing palivizumab on the basis of the risk factors that can make the children eligible for the prophylaxis, the uncertainties related to the initiation and termination of immunoprophylaxis and the correct definition of the risks of the prognostic factors for hospital admission due to RSV infection.

Possible organisational impact in case of hospital –based instead of home –based palivizumab administration to all of the possible children eligible for prophylaxis.

Yes	Uncertain	No
☐	■	☐

Equity
Would inequities be reduced?

The intervention might only be available to those able to pay if it is not covered by insurance/NHS.

Yes	Uncertain	No
☐	■	☐

**Your view of the balance of desirable and undesirable consequences of the intervention**	**Yes**	**Probably**	**Don’t know**	**Probably not**	**No**
	Desirable consequences clearly outweigh undesirable consequences	Desirable consequences probably outweigh undesirable consequences	Consequences equally balanced or uncertain	Undesirable consequences probably outweigh desirable consequences	Undesirable consequences clearly outweigh desirable consequences
**Decision**	**Yes**	**Coverage with evidence development**		**No**	
**Justification** (reason for deciding the intervention should be covered, covered with evidence development or not covered)					
**Implementation** (details regarding the decision, including any restrictions on coverage and conditions for coverage with evidence development)					

References

1) Wang D, Bayliss S, Meads C. Palivizumab for immunoprophylaxis of respiratory syncytial virus (RSV) bronchiolitis in high-risk infants and young children: a systematic review and additional economic modelling of subgroup analyses. Health Technol Assess 2011;15(5).

2) Robinson KA, Odelola OA, Saldanha I, Mckoy N. Palivizumab for prophylaxis against respiratory syncytial virus infection in children with cystic fibrosis. *Cochrane Database of Systematic Reviews* 2010, Issue 2.
