# Supplementary material for: Developing and evaluating communication strategies to support informed decisions and practice based on evidence (DECIDE): protocol and preliminary results
Source: Implement Sci. 2013 Jan 9;8:6. doi: 10.1186/1748-5908-8-6 (PMC3553065; doi:10.1186/1748-5908-8-6)
Supplement: Additional file 3 — Example framework from WP5. [file 1748-5908-8-6-S3.pdf]

Should patients with acute stroke be treated in stroke units, stroke units with early discharge or general medical wards?

Prepared by: Andy Oxman
Date: 4 May 2012

Background

Problem: The organisation of treatment and rehabilitation for acute stroke patients can affect patient outcomes and costs. Many patients in Norway are cared for in general medical wards without a specialised multidisciplinary team that provides care exclusively for stroke patients.

Options: Stroke units are an option where care is provided by nurses, doctors and therapists who specialise in looking after stroke patients and work as a co-ordinated team in a discrete ward caring exclusively for stroke patients. Early supported discharge is an option that aims to get patients back to an active life as quickly as possible. It includes acute treatment in a stroke unit followed by early discharge and follow-up by a multidisciplinary team, coordination of care with primary healthcare providers, and patients living so far as possible at home.

Comparison: Care in an acute medical or neurology ward (general medical wards) without routine multidisciplinary input

Problem: Where best to manage patients with acute stroke
Options: Stroke units with or without early discharge
Comparison: General medical wards

	CRITERIA	JUDGEMENT	EVIDENCE	COMMENTS
PROBLEM	**Is the problem a priority?**	*No* ☐ *Probably not* ☐ *Uncertain* ☐ *Probably* ☐ *Yes* ☑	Acute stroke patients cared for in general medical wards have a high risk of death (27%) and dependency (24%). 15% require institutional care following discharge. [1]	
	**Are a large number of people affected?**	*No* ☐ *Probably not* ☐ *Uncertain* ☐ *Probably* ☐ *Yes* ☑	15,000 strokes per year in Norway. 3rd most common cause of death.<br>Most common cause of serious disability. [2]	

	CRITERIA	JUDGEMENT	EVIDENCE	COMMENTS
BENEFITS & HARMS OF THE OPTIONS	Are the desirable anticipated effects large?	No ☐ Probably not ☐ Uncertain ☐ Probably ☐ Yes ☑	See summary of findings below	
	Are the undesirable anticipated effects small?	No ☐ Probably not ☐ Uncertain ☐ Probably ☐ Yes ☑	See summary of findings below	
	What Is the overall certainty of these anticipated effects?	No included studies ☐ Very low ☐ Low ☐ Moderate ☑ High ☐	See summary of findings below	

***Summary of findings:** Stroke units vs general medical wards* [1]

Outcome (1-12 months)	General wards (per 1000)*	Stroke units (per 1000)	Difference (per 1000) (95% CI)	Certainty of the anticipated effect
Death	265	236	**29 fewer** (from 3 to 53 fewer)	⊕⊕⊕◯ Moderate
Dependency	235	223	**12 fewer** (from 52 fewer to 40 more)	⊕⊕⊕◯ Moderate
Institutionalized	148	117	**31 fewer** (from 58 fewer to 4 more)	⊕⊕◯◯ Low

No adverse effects of stroke units were reported.

*Based on findings in the systematic review. Current risks in Norway are uncertain.

Link to detailed evidence profile

***Summary of findings:** Early supported discharge vs ordinary discharge* [3, 4]

Outcome (1-12 months)	Ordinary discharge (per 1000)	Early Supported discharge (per 1000)	Difference (per 1000) (95% CI)	Certainty of the anticipated effect
Death	236	215	**21 fewer** (from 106 fewer to 120 more)	⊕⊕◯◯ Low
Dependency	223	185	**38 fewer** (from 71 fewer to 2 more)	⊕⊕⊕◯ Moderate
Institutionalized	117	85	**32 fewer** (from 62 fewer to 15 more)	⊕⊕⊕◯ Moderate

No adverse effects of stroke units with early discharge were reported.

*Based on findings in the systematic review of stroke units.

Link to detailed evidence profile

VALUES

Are the desirable effects large relative to undesirable effects?

☐ No ☐ Probably not ☐ Uncertain ☐ Probably ☑ Yes

Quality of life (utility) values for stroke patients:*

Type of stroke	Ara 2008 [5]	Slot 2009 [6]
Mild	0.78	0.93
Moderate	0.61	0.78
Serious	0.47	0.18

*Average values from two studies where 0.00 represents death and 1.00 represents perfect health

RESOURCE USE

Are the resources required small?

☐ No ☐ Probably not ☐ Uncertain ☐ Probably ☑ Yes

Strategy	Total cost per year*: NOK	Total cost per year*: Difference – *Stroke unit versus general ward*	Total cost per year*: Difference – *Early supported discharge versus routine discharge*	Cost per patient [2]: NOK	Cost per patient [2]: Difference – *Stroke unit versus general ward*	Cost per patient [2]: Difference – *Early supported discharge versus ordinary discharge*
General ward)	19 billion			1 270 000		
Stroke unit	14 billion	- 5 billion		933 000	- 337 000	
Stroke unit with early discharge	12 billion		-2 billion	806 000		- 127 000

*Based on 15000 stroke patients per year

Is the incremental cost small relative to the net benefits?

☐ No ☐ Probably not ☐ Uncertain ☐ Probably ☑ Yes

Cost per QALY = -1 million NOK (i.e. a savings of 1 million kroner with each quality adjusted life year saved) for stroke units compared to general wards and -734 000 NOK for stroke units with early discharge compared to stroke units. Sensitivity analyses showed that care in stroke units followed by early supported discharge is the most cost-effective strategy in 88% of the simulations, while care in ordinary stroke units was the most cost-effective in 12% in urban hospitals. [2]

EQUITY

What would be the impact on health inequities?

☐ Increased ☐ Probably increased ☑ Uncertain ☐ Probably reduced ☐ Reduced

Might increase inequities between rural and urban areas

	CRITERIA	JUDGEMENT	EVIDENCE	COMMENTS
ACCEPTABILITY	Is the option acceptable to key stakeholders?	*No* ☐ *Probably not* ☐ *Uncertain* ☐ *Probably* ☑ *Yes* ☐	From a hospital perspective stroke units may cost more (8000 NOK per admission) [2], while communities (not hospitals) benefit from the savings (which occur after discharge from the hospital)	
FEASIBILITY	Is the option feasible to implement?	*No* ☐ *Probably not* ☐ *Uncertain* ☐ *Probably* ☐ *Yes* ☑		- There are stroke units in Norway<br>- It requires space, an initial investment, and a leader to establish a unit<br>- It might not be clear whose responsibility it is to establish a unit

Should patients with acute stroke be treated in stroke units, stroke units with early discharge or general medical wards?

**Balance of consequences**	*Undesirable consequences* clearly outweigh *desirable consequences* ☐	*Undesirable consequences* probably outweigh *desirable consequences* ☐	*Desirable/undesirable consequences* closely balanced or uncertain ☐	*Desirable consequences* probably outweigh *undesirable consequences* ☐	*Desirable consequences* clearly outweigh *undesirable consequences* ☑
**Decision**	***Do not implement the option*** ☐	***Postpone a decision*** ☐	***Do a pilot study*** ☐	***Implement with an impact evaluation*** ☐	***Implement the option*** ☑
	We conclude that patients with acute stroke should be cared for in stroke units with early discharge. All urban hospitals must, therefore, have a stroke unit and communities must have arrangements for early discharge from those units.				
**Justification**	Stroke units with early supported discharge probably will reduce mortality and dependency and save money. The cost-effectiveness analysis suggests that this conclusion is robust.				
**Other implementation considerations**	Implementing this option requires establishing responsibility and accountability for establishing and maintaining stroke units and early discharge, and aligning financial incentives for hospitals and communities; e.g. by compensating hospitals for the costs of establishing and maintaining a stroke unit.				
**Monitoring**	We suggest using the following indicators to monitor the implementation of this decision and inform decisions about the need for further action: establishment of stroke units at all urban hospitals, whether stroke patients are managed in stroke units and discharged early, survival, dependency, institutionalization, hospital costs and costs of community-based health and social services.				
**Evaluation**	Although further evaluation could increase the certainty of the anticipated effects, this is not likely to change the decision. Therefore evaluation of the impacts of this decision is not considered a priority.				

GRADE Evidence Profile: Stroke units versus general medical wards for patients with acute stroke

Author(s): TVR, VHA

Date: 2009-10-16

Question: Should stroke unit *vs.* general ward be used in patients with stroke?

Settings: hospital setting

Bibliography: Organised inpatient (stroke unit) care for stroke (stroke unit trialists' collaboration), 2007

Quality assessment							Summary of findings					
							No of patients		Effect		Quality	Importance
No of studies	Design	Limitations	Inconsistency	Indirectness	Imprecision	Other considerations	stroke unit	general ward	Relative (95% CI)	Absolute		
**Death (follow-up 1-12 months)**												
12	randomised trials[1]	serious[2,3]	no serious inconsistency	no serious indirectness	no serious imprecision	none	431/1888 (22.8%)	488/1840 (26.5%)	RR 0.89 (0.8 to 0.99)	29 fewer per 1000 (from 3 fewer to 53 fewer)	⊕⊕⊕O MODERATE	CRITICAL
**dependency (follow-up 6-12 months)**												
9	randomised trials[4]	serious[3,5]	no serious inconsistency	no serious indirectness	no serious imprecision	none	337/1373 (24.5%)	323/1375 (23.5%)	RR 0.95 (0.78 to 1.17)	12 fewer per 1000 (from 52 fewer to 40 more)	⊕⊕⊕O MODERATE	CRITICAL
**Institutionalized (follow-up 1-12 months)**												
11	randomised trials[6]	serious[3,7]	no serious inconsistency	no serious indirectness	serious[8]	none	250/1857 (13.5%)	266/1802 (14.8%)	RR 0.79 (0.61 to 1.03)	31 fewer per 1000 (from 58 fewer to 4 more)	⊕⊕OO LOW	CRITICAL

[1] 9 of studies were RCT, 3 was controlled clinical trial (incl. some form of random allocation: e.g. bed availability, date of admission)

[2] 9 of 12 studies were RCT, 3 of them with adequate allocation, 6 of them with unclear allocation concealment

[3] Blinding of patients and physicians not possible. Only 4 studies had blinded assessors/ follow-up. However, end -points were mainly objective and therefore less prone to bias than if they had been more subjective. Even so we chose to downgrade.

[4] 7 of studies were RCT, 2 was controlled clinical trial (incl. some form of random allocation: e.g. bed availability ,date of admission)

[5] 7 of 9 studies were RCT, 2 of them with adequate allocation, 5 of them with unclear allocation concealment

[6] 8 of studies were RCT, 3 was controlled clinical trial (incl. some form of random allocation: e.g. bed availability ,date of admission)

[7] 8 of 11 studies were RCT, 2 of them with adequate allocation, 6 of them with unclear allocation concealment

[8] 95% confidence interval (or alternative estimate of precision) around the pooled or best estimate of effect includes both 1) no effect and 2) appreciable benefit or appreciable harm. GRADE suggests that the threshold for "appreciable benefit" or "appreciable harm" that should be considered for downgrading is a relative risk reduction (RRR) or relative risk increase (RRI) greater than 25%.

(Return to decision framework)

GRADE Evidence Profile: Early supported discharge versus ordinary discharge for patients with acute stroke

Author(s): TVR + VHA

Date: 2009-10-16

Question: Should early supported discharge *vs.* ordinary discharge be used in patients with acute stroke?

Settings: hospital setting and rehabilitation

Bibliography: Services for reducing duration of hospital care for acute stroke patients (early supported discharge trialists) + Askim et al., 2004

Quality assessment							Summary of findings					Importance
							No of patients		Effect		Quality	
No of studies	Design	Limitations	Inconsistency	Indirectness	Imprecision	Other considerations	early supported discharge	ordinary discharge	Relative (95% CI)	Absolute		
**Death (follow-up 3-12 months)**												
6	randomised trials	no serious limitations[1]	no serious inconsistency	no serious indirectness	very serious[2,3,4]	none	27/376 (7.2%)	30/370 (8.1%)	RR 0.91 (0.55 to 1.51)	7 fewer per 1000 (from 36 fewer to 41 more)	⊕⊕OO LOW	CRITICAL
**Dependency (follow-up 3-12 months)**												
5	randomised trials	no serious limitations[1]	no serious inconsistency	no serious indirectness	serious[3]	none	112/345 (32.5%)	133/339 (39.2%)	RR 0.83 (0.68 to 1.01)	67 fewer per 1000 (from 126 fewer to 4 more)	⊕⊕⊕O MODERATE	CRITICAL
**Institutionalized (follow-up 3-12 months)**												
3	randomised trials	no serious limitations[1]	no serious inconsistency	no serious indirectness	serious[2,3]	none	29/244 (11.9%)	39/241 (16.2%)	RR 0.73 (0.47 to 1.13)	44 fewer per 1000 (from 86 fewer to 21 more)	⊕⊕⊕O MODERATE	CRITICAL

[1] Blinding of patients and physicians was not possible. However studies used blinded assessors/ follow-up.

[2] Total number of events is less than 300 (a threshold rule-of-thumb value) (based on: Mueller et al. Ann Intern Med. 2007;146:878-881 <http://www.annals.org/cgi/content/abstract/146/12/878>)

[3] 95% confidence interval (or alternative estimate of precision) around the pooled or best estimate of effect includes both 1) no effect and 2) appreciable benefit or appreciable harm. GRADE suggests that the threshold for "appreciable benefit" or "appreciable harm" that should be considered for downgrading is a relative risk reduction (RRR) or relative risk increase (RRI) greater than 25%.

[4] Estimate and confidence interval include both appreciable benefit and appreciable harm for the intervention.

(Return to decision framework)

Explanations

Criteria	Question	Explanation
**Is the problem a priority?**<br><br>(Return to decision framework)	*Are the consequences of the problem serious (i.e. severe or important in terms of the potential benefits or savings)? Is the problem urgent? Is it a recognised priority (e.g. based on a national health plan)?*	The more serious a problem is, the more likely it is that an option that addresses the problem will be a priority (e.g. diseases that are fatal or disabling are likely to be a higher priority than diseases that only cause minor distress)
**Are a large number of people affected?**<br><br>(Return to decision framework)	*Are a large number of people affected by the problem?*	The more people who are affected, the more likely it is that an option that addresses the problem will be a priority
**Are the desirable anticipated effects large?**<br><br>(Return to decision framework)	*Are the desirable anticipated effects (including health and other benefits) of the option large (taking into account the severity or importance of the desirable consequences and the number of people affected)?*	The larger the desirable effects (benefits), including non-health outcomes, the more likely it is that an option will be a priority. Consideration should be given to subgroups (different effects in different populations) and to differences in the baseline risk (the risk in the comparison group)
**Are the undesirable anticipated effects small?**<br><br>(Return to decision framework)	*Are the undesirable effects (including adverse health effects and other harms) of the option small (taking into account the severity or importance of the adverse effects and the number of people affected)?*	The greater the risk of undesirable effects (harms), the less likely it is that an option will be a priority
**What Is the overall certainty of these anticipated effects?**<br><br>(Return to decision framework)	*What is the overall certainty of the anticipated effects, across all of the outcomes that are critical to making a decision?*	The less the certainty in the anticipated impacts, the less likely that an option will be a priority (or the more important it is likely to be to conduct a pilot study or impact evaluation)
**Are the desirable effects large relative to undesirable effects?**<br><br>(Return to decision framework)	*Are the desirable anticipated effects (benefits) large relative to the undesirable anticipated effects (harms)?*	The larger the desirable effects in relation to the undesirable effects, taking into account the values of those affected (i.e. the relative value they attach to the desirable and undesirable outcomes), the more likely it is that an option will be a priority
**Are the resources required small?**<br><br>(Return to decision framework)	*Would the option require a small investment of resources or save resources?*	The greater the cost, the less likely it is that an option will be a priority
**Is the incremental cost small relative to the net benefits?**<br><br>(Return to decision framework)	*Is the cost small relative to the net benefits (benefits minus harms)?*	The lower the cost per unit of benefit, the more likely it is that an option will be a priority (From a societal perspective, taking into account the robustness of the estimate (sensitivity analyses) and the timing of the benefits, harms and costs)
**Impacts on equity**<br><br>(Return to decision framework)	*Would the option reduce health inequities?*	Policies or programmes that reduce inequities may be more of a priority than ones that do not (or ones that increase inequities)

Criteria	Question	Explanation
**Is the option acceptable to key stakeholders?**<br>(Return to decision framework)	*Are key stakeholders likely to find the option acceptable (given the relative importance they attach to the desirable and undesirable consequences of the option; the timing of the benefits, harms and costs; and their moral values)?*	The less acceptable an option is to key stakeholders, the less likely it is to be a priority, taking into account:<br>- Who benefits (or is harmed) and who pays (or saves)<br>- When the benefits, adverse effects, and costs occur (and the discount rates of key stakeholders; e.g. politicians may have a high discount rate for anything that occurs beyond the next election)<br>Unacceptability may be due to some stakeholders<br>- Attaching more value (relative importance) to the undesirable consequences than to the desirable consequences or costs of an option (either because of how they might be affected personally or because of their perceptions of the relative importance of consequences for others)<br>- Unwillingness to accept costs or undesirable effects in the short term for desirable effects in the future<br>- Moral disapproval (i.e. in relationship to ethical principles such as autonomy, nonmaleficence, beneficence or justice)
**Is the option feasible to implement?**<br>(Return to decision framework)	*Can the option be accomplished or brought about?*	The less feasible (capable of being accomplished or brought about) an option is, the less likely it is that it will be a priority (i.e. the more barriers there are that would be difficult to overcome)

	Question	Explanation
**Balance of consequences**<br>(Return to decision framework)	*What is the balance between the desirable and undesirable consequences?*	Based on the evidence presented, do the desirable consequences outweigh the undesirable consequences, or vice versa?
**Decision**<br>(Return to decision framework)	*Based on the balance of the consequences and the other criteria in the framework, what is the decision?*	Select one decision option and state the decision in plain language, including important qualifications
**Justification**<br>(Return to decision framework)	*What is the justification for the decision, based on the criteria in the framework that drove the decision?*	Summarise the justification for the decision based on the criteria in the framework that drove the decision
**Other implementation considerations**<br>(Return to decision framework)	*What other factors (besides those addressed by the qualifications) should be considered when implementing the decision, including strategies to address concerns about acceptability and feasibility?*	Summarise important implementation considerations other than those addressed by the qualifications attached to the decision, including strategies to address concerns about acceptability, feasibility, the timeframe, who is responsible and accountability?
**Monitoring**<br>(Return to decision framework)	*What indicators should be monitored?*	Identify any important indicators that should be monitored when the decision is implemented
**Evaluation**<br>(Return to decision framework)	*Is there a need to evaluate the impacts of the decision, either in a pilot study or an impact evaluation carried out alongside of full implementation of the decision?*	Identify any needs for a pilot study or impact evaluation

GRADE ratings for certainty of anticipated effect (also called "quality of evidence" or "confidence in the estimates")

⊕⊕⊕⊕	**High**	It is very likely that the effect will be close to what was found in the research.
⊕⊕⊕◯	**Moderate**	It is likely that the effect will be close to what was found in the research, but there is a possibility that it will be substantially different.
⊕⊕◯◯	**Low**	It is likely that the effect will be substantially different from what was found in the research, but the research provides an indication of what might be expected.
⊕◯◯◯	**Very low**	The anticipated effect is very uncertain and the research does not provide a reliable indication of what might be expected.

(Return to decision framework)

References

1. Stroke Unit Trialist Collaboration. Organised inpatient (stroke unit) care for stroke. Cochrane Database of Systematic Reviews 2007, Issue 4: CD000197.

2. Hamidi V, Wisløff T, Ringerike T, Linnestad KK, Harboe I, Klemp M. Behandling av pasienter med akutt hjerneslag i slagenheter (med og uten tidlig støttet utskriving). Rapport fra Kunnskapssenteret nr 18-2010. Oslo: Nasjonalt kunnskapssenter for helsetjenesten, 2010.

3. Early Supported Discharge Trialists. Services for reducing duration of hospital care for acute stroke patients. Cochrane Database of Systematic Reviews 2005; Issue 2: CD000443.

4. Askim T, Rohweder G, Lydersen S, Indredavik B. Evaluation of an extended stroke unit service with early supported discharge for patients living in a rural community. A randomized controlled trial. Clin Rehabil 2004; 18(3):238-48.

5. Ara R, Tumur I, Pandor A, Duenas A, Williams R, Wilkinson A, et al. Ezetimibe for the treatment of hypercholesterolaemia: a systematic review and economic evaluation. Health Technol Assess 2008; 12(21):iii,xi-iii,212.

6. Slot KB, Berge E. Thrombolytic treatment for stroke: patient preferences for treatment, information, and involvement. J Stroke Cerebrovasc Dis 2009; 18(1):17-22.
