# Supplementary material for: Developing and evaluating communication strategies to support informed decisions and practice based on evidence (DECIDE): protocol and preliminary results
Source: Implement Sci. 2013 Jan 9;8:6. doi: 10.1186/1748-5908-8-6 (PMC3553065; doi:10.1186/1748-5908-8-6)
Supplement: Additional file 4 — The Guideline Development Tool. [file 1748-5908-8-6-S4.pdf]

The Guideline Development Tool

The Guideline Development Tool, an interactive, browser based tool (www.guidelinedevelopment.org) will include the following functionality in addition to the current GRADEpro features:

- Learning modules about guideline development
- Multiple participants can collaborate offline through a browser based application
- Individuals can work online and offline
- Independent of the operating system (Windows and Mac OS)
- Interaction with Cochrane Collaboration's Review Manager (RevMan) program
- Assessment of existing guidelines and systematic reviews for their credibility
- Export of evidence syntheses to Database of Evidence Profiles (separate online repository of “evidence tables” – see below)
- Communicate with a dedicated website that will serve as an interface between the software and multiple people providing input (e.g. guideline panel members); it will generate forms on a secure website enabling people, e.g. to vote, and then collect the results of voting
- Simple text editor allowing to produce basic text formatting + tables, insert pictures, add footnotes and explanations, tracking changes with name of the user and time, inserting citations from a local citation manager
- Basic citation manager capable of importing from PubMed
- Ability to create templates to produce various documents based on those templates partially automatically filled-in with information entered into pre-designed forms (several different forms might feed into one document) and partly by entering the information directly into the document
- Produce interactive Summary of Findings Tables (SoF)

The modules of the Guideline Development Tool will include, among others:

1. Address book
2. Conflict of interest (COI) management
3. Topic proposal and selection
4. Developing the scope
5. Generation of structured questions (PICO)
6. Identifying outcomes of interest for each question

7. Evidence retrieval - identifying existing guidelines and systematic reviews and assessing them for their credibility
8. Reference management
9. Data extraction
10. Word processor
11. GRADEprofiler
12. A toolbox for the development of derivatives
